# Out-of-Hours Appendicectomy Is Associated With Earlier Theatre Access and Comparable Clinical Outcomes in a UK Tertiary Centre

**DOI:** 10.7759/cureus.115055

**Published:** 2026-08-23

**Authors:** Zlatin Milanov, Catherine McKeever, Thomas Curl-Roper

**Affiliations:** 1 Emergency General Surgery, Norfolk and Norwich University Hospital, Norwich, GBR; 2 General Surgery, Norfolk and Norwich University Hospital, Norwich, GBR

**Keywords:** acute appendicitis，laparoscopic appendectomy，uncomplicated acute appendicitis, adverse clinical outcomes, emergency appendicectomy, general emergency surgery, laparoscopic emergency surgery, length of hospital stay (los), operative timing of appendicitis, out-of-hours surgery, time-to-theatre

## Abstract

Background

Acute appendicitis is one of the most common surgical emergencies and a frequent indication for emergency abdominal surgery. Concerns regarding reduced staffing, competing emergency workload and limited overnight theatre availability have contributed to ongoing debate regarding outcomes following surgery outside routine working hours. Contemporary UK tertiary-centre data remain limited. This study evaluated the association between operative timing, theatre access and short-term clinical outcomes following emergency appendicectomy.

Methods

A retrospective cohort study was conducted at a UK tertiary centre, including all adults undergoing emergency appendicectomy between January and June 2025. Patients were categorised according to operative start time as in-hours (08:00-17:30) or out-of-hours (17:31-07:59). Data included age, American Society of Anesthesiologists (ASA) Physical Status Classification score, imaging-defined disease severity, time from radiological diagnosis to theatre, length of hospital stay (LOS), postoperative complications and 30-day hospital re-presentations. Continuous variables were assessed for normality using the Shapiro-Wilk test and compared using the Mann-Whitney U test. Categorical variables were compared using Pearson's chi-square test or Fisher's exact test where appropriate.

Results

A total of 127 patients were included: 69 in-hours and 58 out-of-hours. Mean age was 43.2 ± 18.2 and 44.9 ± 19.3 years, respectively (Mann-Whitney U = 1900.5, p = 0.628). Median ASA score was 2 (interquartile range (IQR) 1-2) in both cohorts (p = 0.686). Imaging-defined complicated appendicitis occurred in 32/69 (46.4%) and 28/58 (48.3%) patients, respectively (p = 0.972).

Mean time from radiological confirmation to surgery was 22.9 ± 24.4 hours in-hours and 16.0 ± 14.0 hours out-of-hours (Mann-Whitney U = 2372.0, p = 0.073). Median LOS was 2 (IQR 1-4) and 2 (IQR 1-3) days, respectively (p = 0.430). Postoperative complications occurred in 11/69 (15.9%) and 12/58 (20.7%) patients, respectively (p = 0.489), while 30-day hospital re-presentations occurred in 9/69 (13.0%) and 7/58 (12.1%), respectively (p = 0.869). Overall, 23/127 (18.1%) patients experienced at least one postoperative complication; intra-abdominal collection was the most common, occurring in 11/127 (8.7%). Sixteen patients (12.6%) re-presented within 30 days, including seven hospital readmissions and nine Surgical Same Day Emergency Care (SDEC) reviews. Two patients required conversion to open right hemicolectomy for severe complicated appendicitis with caecal involvement.

Conclusions

No statistically significant differences were observed between in-hours and out-of-hours appendicectomy in time from radiological confirmation to surgery, postoperative complications, LOS or 30-day hospital re-presentation. Within this cohort, no difference in measured short-term outcomes was detected according to operative timing. Larger multicentre studies are required to assess whether these findings are consistent across different emergency surgical settings and to account for theatre availability, staffing and case prioritisation.

## Introduction

Acute appendicitis remains one of the most common surgical emergencies worldwide, with a lifetime risk of approximately 7-8%, and is a leading indication for emergency abdominal surgery [1]. Early appendicectomy remains the standard treatment for most patients with acute appendicitis, and the current World Society of Emergency Surgery (WSES) Jerusalem Guidelines recommend operative management within 24 hours for clinically stable patients to minimise morbidity while avoiding unnecessary delays [1].

Historically, prompt appendicectomy has been advocated to reduce the risk of perforation, diffuse peritonitis, intra-abdominal sepsis and prolonged hospitalisation. Consequently, delays in operative management have traditionally been viewed with concern [2,3]. However, increasing pressure on emergency surgical services, limited theatre capacity and competing emergency workloads have prompted greater interest in whether operative timing is associated with short-term clinical outcomes.

Emergency appendicectomy performed outside routine working hours has also raised concerns regarding reduced overnight staffing, limited consultant availability and competing emergency theatre demands. Nevertheless, contemporary evidence has challenged the assumption that operative timing is associated with poorer outcomes. A recent systematic review and meta-analysis by Tang et al. reported no statistically significant differences in postoperative complications, mortality, length of hospital stay or readmission rates between patients undergoing daytime and nighttime appendicectomy [4]. Similarly, the WSES Jerusalem Guidelines recommend that operative timing should be guided by clinical urgency rather than restricted according to the time of day, provided surgery is performed within the recommended timeframe [1].

Despite these findings, important evidence gaps remain. Published studies have been conducted across diverse healthcare systems and emergency surgical services, making it difficult to determine the applicability of their findings to individual UK hospitals. Contemporary evidence from UK tertiary centres remains limited, particularly within the National Health Service (NHS). As emergency theatre access, staffing models and service provision vary between institutions, local evaluation remains important to determine whether operative timing is associated with theatre access and short-term clinical outcomes.

Therefore, this study aimed to evaluate the association between operative timing and theatre access and short-term clinical outcomes following emergency appendicectomy at a UK tertiary centre. Specifically, outcomes following in-hours (IH) and out-of-hours (OH) appendicectomy were compared with respect to time to theatre, length of hospital stay, postoperative complications and 30-day hospital re-presentation rates.

## Materials and methods

Study design and setting

A retrospective observational cohort study was conducted at Norfolk and Norwich University Hospital (NNUH), a tertiary centre providing emergency general surgical services to a large regional population in the East of England. The study evaluated the association between operative timing, theatre access and short-term clinical outcomes following emergency appendicectomy.

Patient selection

All consecutive adult patients (aged ≥18 years) undergoing emergency appendicectomy between 1 January and 30 June 2025 were identified using the hospital's electronic theatre management system and operative database. Patient records were subsequently reviewed through the electronic patient record system.

Patients were included if they underwent emergency appendicectomy with pre-operative imaging confirming acute appendicitis. Patients were excluded if they underwent elective appendicectomy, were managed non-operatively, or had incomplete electronic clinical records that prevented assessment of the study outcomes.

Cohort allocation

Patients were allocated to study groups according to the recorded start time of surgery rather than the time of hospital admission. Operative start time was selected because it reflected the operative environment, including theatre availability, staffing levels, consultant supervision and emergency surgical service provision.

Patients were categorised according to the recorded start time of surgery as IH, defined as operations commencing between 08:00 and 17:30, or OH, defined as operations commencing between 17:31 and 07:59.

Data collection

Clinical data were collected retrospectively from multiple electronic clinical systems, including Integrated Clinical Environment (ICE) for discharge summaries and imaging reports, ORSOS (Operating Room Scheduling Office System) for theatre records, MetaVision for review of original operative notes and clinical documentation, Synapse for radiological imaging, and Surgical Same Day Emergency Care (SDEC) documentation for follow-up and 30-day re-presentation data. Where discrepancies were identified between electronic sources, the original operative notes and clinical documentation available through MetaVision were reviewed to verify the recorded data.

Collected demographic variables included patient age and sex. Clinical variables included the ASA Physical Status Classification score, imaging-defined disease severity, imaging modality, operative timing, time from radiological diagnosis to commencement of surgery, length of hospital stay, operative approach, conversion to open surgery, conversion to right hemicolectomy, postoperative complications occurring within 30 days, 30-day hospital re-presentations and 30-day hospital readmissions.

Baseline physiological status was assessed using the ASA Physical Status Classification System [5].

All patients had imaging-confirmed acute appendicitis prior to operative intervention. Pre-operative imaging comprised computed tomography (CT) in 112/127 (88.2%) patients, ultrasound in 14/127 (11.0%) patients and magnetic resonance imaging (MRI) in 1/127 (0.8%) patient.

Complicated appendicitis was defined according to pre-operative imaging demonstrating perforation, peri-appendiceal abscess, inflammatory phlegmon or diffuse peritonitis, consistent with the WSES Jerusalem Guidelines for the management of acute appendicitis [1].

Operative approach

The operative approach and any conversion to open surgery were recorded from the operative documentation. All procedures were commenced laparoscopically. Two patients required conversion to open right hemicolectomy because of severe complicated appendicitis with caecal involvement.

Outcome measures

The primary outcomes were postoperative complications and length of hospital stay. Secondary outcomes included time from radiological diagnosis to commencement of surgery, 30-day hospital re-presentation and 30-day hospital readmission.

Time to theatre was calculated as the interval between the documented time of radiological confirmation of acute appendicitis and the recorded start time of surgery. Radiological confirmation was used as the starting point because all patients had imaging-confirmed appendicitis before operative intervention.

Postoperative complications were defined as adverse clinical events occurring within 30 days following appendicectomy that required clinical assessment or treatment. Complications were identified through review of electronic clinical documentation, discharge summaries, operative records and SDEC records.

Complications were classified according to the Clavien-Dindo classification system based on the treatment required. Grade I complications were defined as deviations from the normal postoperative course not requiring pharmacological, radiological or operative intervention, with the exception of treatments permitted within the Grade I classification. Complications requiring pharmacological treatment beyond these Grade I measures, including antibiotic therapy, were classified as Grade II. No patients required radiological drainage, further operative intervention for a postoperative complication, intensive care management or treatment associated with Clavien-Dindo Grade III-V complications.

Statistical analysis

Data were entered and analysed using Microsoft Excel for Microsoft 365 (Microsoft Corporation, Redmond, WA, USA). Normality of continuous variables was assessed using the Shapiro-Wilk test. Continuous variables were summarised as means with standard deviations and/or medians with interquartile ranges (IQRs), as appropriate. As the continuous variables assessed did not demonstrate a normal distribution, comparisons between groups were performed using the Mann-Whitney U test.

Categorical variables were summarised as frequencies and percentages and compared using Pearson's chi-square (χ²) test or Fisher's exact test where appropriate. A two-sided p-value <0.05 was considered statistically significant.

No formal prospective sample-size or power calculation was performed because this was a retrospective cohort comprising all eligible patients during the predefined study period.

Missing data

Data completeness was assessed for all study variables. Patients with incomplete electronic clinical records that prevented assessment of the study outcomes were excluded according to the predefined eligibility criteria. Where individual variables were unavailable, analyses were performed using available data and no statistical imputation was performed.

Ethics statement

This retrospective observational study was registered with the Norfolk and Norwich University Hospital Clinical Audit Department. The audit registration number was not available to the authors at the time of manuscript submission. The study was conducted using anonymised routinely collected clinical data. As no alteration to patient management occurred and only retrospective anonymised data were analysed, formal NHS Research Ethics Committee approval was not required.

## Results

**Figure 1:**
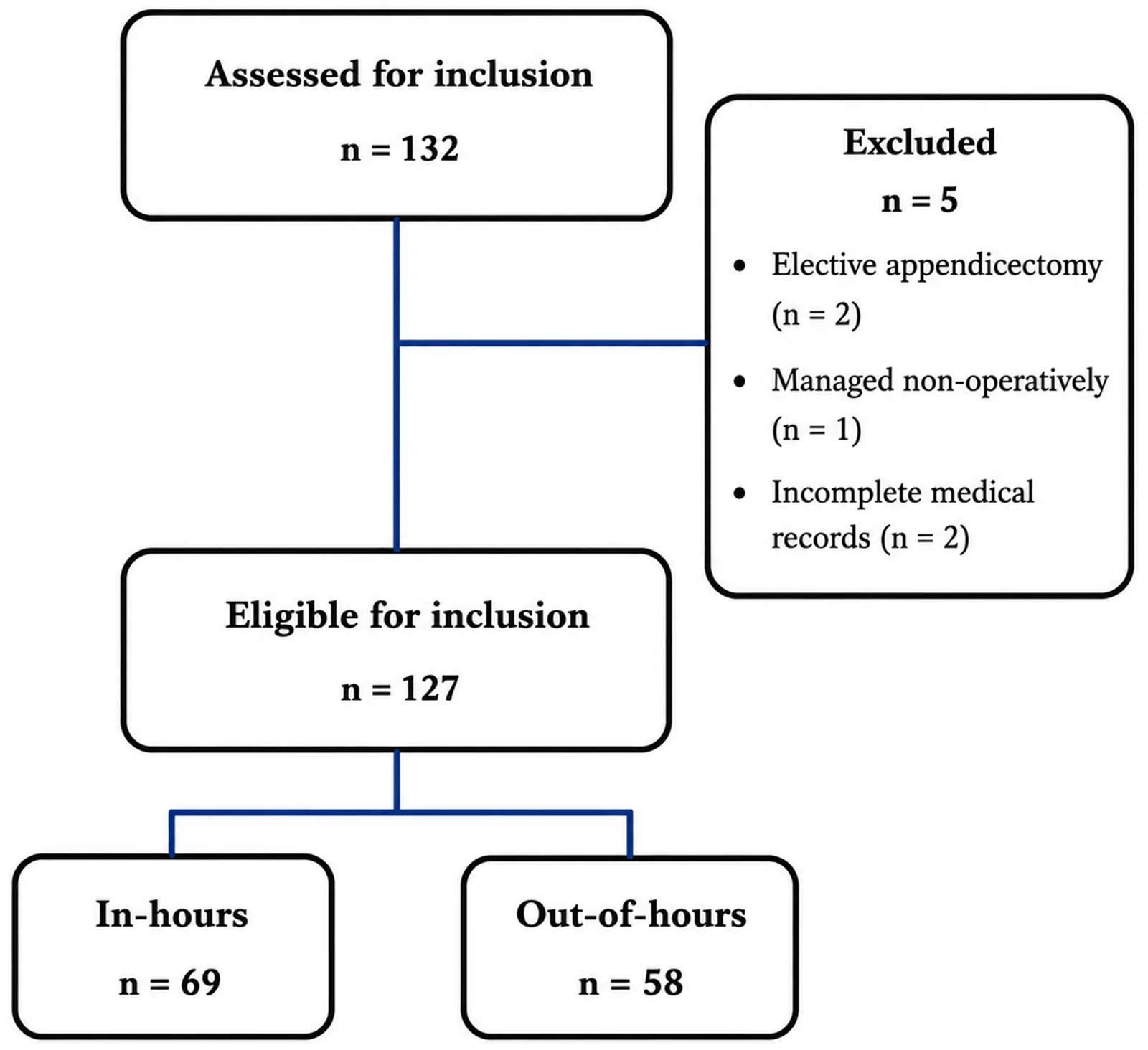
STROBE Flow Diagram of Patient Selection Flow diagram demonstrating patient selection and cohort allocation. A total of 132 adult patients undergoing emergency appendicectomy between January and June 2025 were identified. Following application of the predefined eligibility criteria, five patients were excluded, leaving 127 patients for analysis. Patients were subsequently categorised according to operative start time into the in-hours (08:00-17:30; n = 69) and out-of-hours (17:31-07:59; n = 58) cohorts. Image credit: The authors, using ChatGPT (OpenAI, San Francisco, CA, USA). The authors are responsible for the scientific integrity of this figure.

A total of 132 adult patients who underwent emergency appendicectomy between 1 January and 30 June 2025 were identified through the hospital's electronic theatre management system. Following application of the predefined eligibility criteria, five patients were excluded, resulting in a final study cohort of 127 patients for analysis (Figure 1).

Baseline demographic and disease characteristics are summarised in Table 1. Mean age was 43.2 ± 18.2 years in the IH cohort and 44.9 ± 19.3 years in the OH cohort (Mann-Whitney U = 1900.5, p = 0.628). Median ASA Physical Status Classification score was 2 (IQR 1-2) in both cohorts (Mann-Whitney U = 1928.5, p = 0.686). Imaging-defined complicated appendicitis was identified in 32/69 (46.4%) patients undergoing IH appendicectomy and 28/58 (48.3%) undergoing OH appendicectomy (χ² = 0.001, p = 0.972).

**Table 1:** Baseline Demographic and Disease Characteristics of Patients Undergoing In-Hours and Out-of-Hours Appendicectomy Continuous variables are presented as mean ± standard deviation or median (interquartile range), as appropriate. Group comparisons were performed using the Mann-Whitney U test and Pearson's chi-square (χ²) test. ASA, American Society of Anesthesiologists; IQR, interquartile range. Image credit: The authors, using ChatGPT (OpenAI, San Francisco, CA, USA). The authors are responsible for the scientific integrity of this table.

Characteristic	In-hours (n = 69)	Out-of-hours (n = 58)	Statistical test	Test statistic	p-Value
Mean age, years	43.2 ± 18.2	44.9 ± 19.3	Mann-Whitney U test	U = 1900.5	0.628
Median ASA score (IQR)	2 (1-2)	2 (1-2)	Mann-Whitney U test	U = 1928.5	0.686
Complicated appendicitis, n (%)	32 (46.4%)	28 (48.3%)	Pearson χ² test	χ² = 0.001	0.972

Operative outcomes

Operative timing and short-term clinical outcomes are summarised in Table 2. Mean time from radiological confirmation of appendicitis to commencement of surgery was 22.9 ± 24.4 hours in the IH cohort and 16.0 ± 14.0 hours in the OH cohort (Mann-Whitney U = 2372.0, p = 0.073). Median length of stay was 2 (IQR 1-4) days in the IH cohort and 2 (IQR 1-3) days in the OH cohort (Mann-Whitney U = 2155.0, p = 0.430).

**Table 2:** Comparison of Operative Timing and Clinical Outcomes Between In-Hours and Out-of-Hours Appendicectomy Continuous variables are presented as mean ± standard deviation or median (interquartile range), as appropriate. Group comparisons were performed using the Mann-Whitney U test and Pearson's chi-square (χ²) test. LOS, length of stay; IQR, interquartile range. Image credit: The authors, using ChatGPT (OpenAI, San Francisco, CA, USA). The authors are responsible for the scientific integrity of this table.

Outcome	In-hours (n = 69)	Out-of-hours (n = 58)	Statistical test	Test statistic	p-Value
Time to theatre, hours (mean)	22.9 ± 24.4	16.0 ± 14.0	Mann-Whitney U test	U = 2372.0	0.073
Median length of stay, days (IQR)	2 (1-4)	2 (1-3)	Mann-Whitney U test	U = 2155.0	0.430
Postoperative complications, n (%)	11 (15.9%)	12 (20.7%)	Pearson χ² test	χ² = 0.479	0.489
Thirty-day hospital re-presentations, n (%)	9 (13.0%)	7 (12.1%)	Pearson χ² test	χ² = 0.027	0.869

Postoperative complications occurred in 11/69 (15.9%) patients in the IH cohort and 12/58 (20.7%) patients in the OH cohort (χ² = 0.479, p = 0.489). Thirty-day hospital re-presentations occurred in 9/69 (13.0%) and 7/58 (12.1%) patients, respectively (χ² = 0.027, p = 0.869).

Postoperative complications

**Figure 2:**
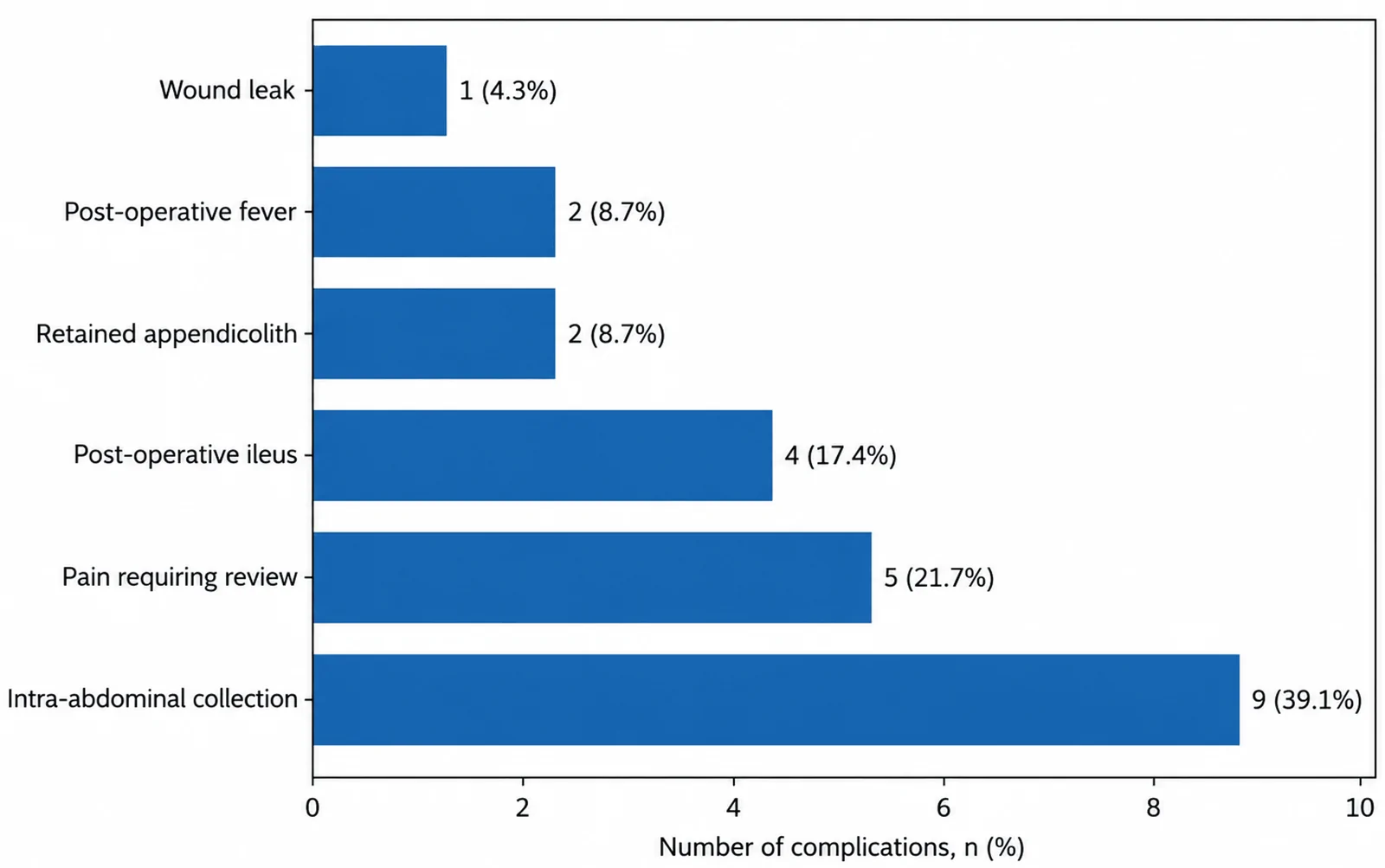
Distribution of Postoperative Complications Following Emergency Appendicectomy Distribution of postoperative complications among the 23 patients who experienced at least one postoperative complication following emergency appendicectomy. Intra-abdominal collections were the most common complication. All collections were managed with antibiotics alone, with no patient requiring image-guided drainage or further operative intervention. Pain requiring review was managed with analgesia, postoperative ileus was managed conservatively, postoperative fever was managed without additional intervention, and the wound leak was managed with antibiotics. Image credit: The authors, using ChatGPT (OpenAI, San Francisco, CA, USA). The authors are responsible for the scientific integrity of this figure.

The distribution of postoperative complications is illustrated in Figure 2. Overall, 23/127 (18.1%) patients experienced at least one postoperative complication. Intra-abdominal collection was the most common complication, occurring in 11/127 (8.7%) patients, followed by pain requiring review in 5/127 (3.9%), postoperative ileus in 4/127 (3.1%), postoperative fever in 2/127 (1.6%) and wound leak in 1/127 (0.8%). The two cases involving a retained appendicolith were managed as intra-abdominal collections with antibiotics and are therefore included within the collection category.

All intra-abdominal collections were managed with antibiotics alone, with no patient requiring image-guided drainage or further operative intervention. Pain requiring review was managed with analgesia. Postoperative ileus was managed conservatively, postoperative fever was managed without additional intervention, and the wound leak was managed with antibiotics.

Postoperative complication rates were 11/69 (15.9%) in the IH cohort and 12/58 (20.7%) in the OH cohort (χ² = 0.479, p = 0.489).

Thirty-day re-presentations

**Figure 3:**
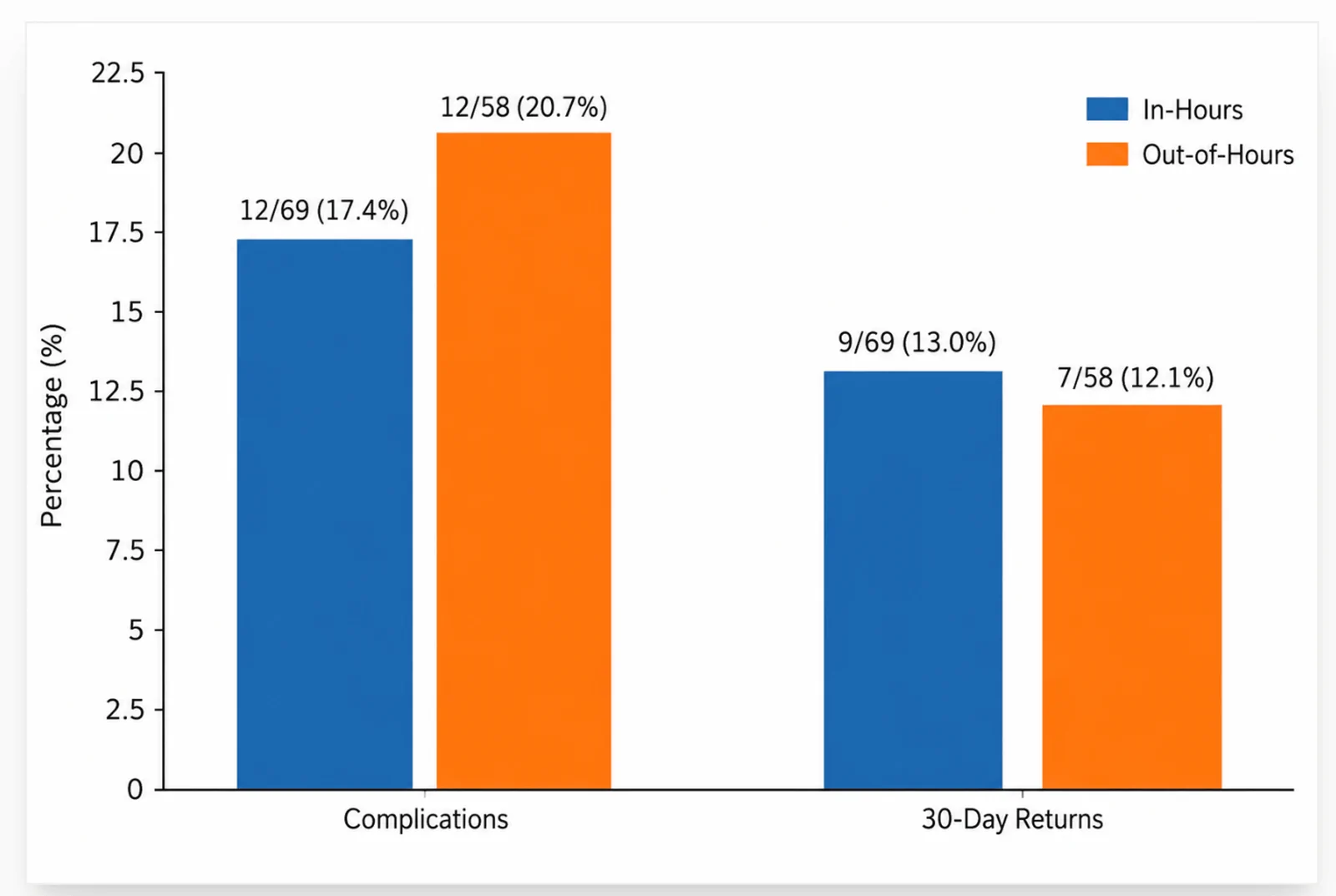
Comparison of Postoperative Complication and 30-Day Hospital Re-Presentation Rates Between In-Hours and Out-of-Hours Appendicectomy Comparison of postoperative complication rates and 30-day hospital re-presentation rates between in-hours and out-of-hours appendicectomy cohorts. Postoperative complications occurred in 11/69 (15.9%) in-hours and 12/58 (20.7%) out-of-hours patients, while 30-day hospital re-presentations occurred in 9/69 (13.0%) and 7/58 (12.1%), respectively. Image credit: The authors, using ChatGPT (OpenAI, San Francisco, CA, USA). The authors are responsible for the scientific integrity of this figure.

Thirty-day re-presentation outcomes are summarised in Figure 3. Overall, 16/127 (12.6%) patients re-presented to hospital within 30 days of discharge. Seven patients required hospital readmission, while nine were managed through the SDEC pathway without requiring hospital admission.

Thirty-day hospital re-presentations occurred in 9/69 (13.0%) patients undergoing IH appendicectomy and 7/58 (12.1%) undergoing OH appendicectomy (χ² = 0.027, p = 0.869). The most common reasons for re-presentation were postoperative pain, wound-related complications and intra-abdominal collections.

Conversion to right hemicolectomy

Two patients required conversion to open right hemicolectomy because of severe complicated appendicitis with caecal involvement. Both cases occurred in the IH cohort and were performed with consultant surgeons present in theatre, facilitating senior intraoperative decision-making.

The first patient was a 62-year-old female with an appendiceal mass and perforation extending into the anterior abdominal wall, associated with poor caecal visualisation and concern for caecal involvement. A right hemicolectomy with primary ileocolic anastomosis was performed.

The second patient was a 23-year-old male presenting in septic shock with four-quadrant faeculent peritonitis, extensive caecal perforation and no identifiable appendix. Owing to profound physiological instability requiring inotropic support, a right hemicolectomy without primary anastomosis was initially performed, followed by planned re-look laparotomy and delayed ileocolic anastomosis the following day.

Both patients subsequently developed intra-abdominal collections, which were managed conservatively with intravenous antibiotics without image-guided drainage or further operative intervention.

## Discussion

Principal findings

This study evaluated the association between operative timing and theatre access and short-term outcomes following emergency appendicectomy at a UK tertiary centre. Mean time from radiological confirmation of appendicitis to commencement of surgery was 22.9 ± 24.4 hours in the IH cohort and 16.0 ± 14.0 hours in the OH cohort (Mann-Whitney U = 2372.0, p = 0.073). No statistically significant differences were identified between cohorts in postoperative complications, length of hospital stay or 30-day hospital re-presentation rates. Within this cohort, therefore, no difference in the measured short-term clinical outcomes was detected according to operative timing. However, the retrospective observational design and relatively modest sample size limit the extent to which these findings can be interpreted as demonstrating equivalence between operative periods.

Comparison with existing literature

Historically, concerns have been raised regarding emergency surgery performed outside routine working hours because of reduced overnight staffing, limited consultant availability, fatigue and competing emergency theatre demands [4,6,7]. Earlier studies also suggested that delays to appendicectomy may increase the risk of perforation and other adverse outcomes, supporting prompt operative intervention [2,3]. However, more recent evidence has challenged the assumption that surgery performed outside routine working hours is associated with poorer clinical outcomes.

A recent systematic review and meta-analysis by Tang et al. reported no statistically significant differences in postoperative complications, mortality, length of hospital stay or readmission rates between patients undergoing daytime and nighttime appendicectomy [4]. Similarly, Shen et al. reported no statistically significant association between operative timing and mortality, postoperative complications or hospital length of stay [8]. Mönttinen et al. reported comparable postoperative morbidity and mortality following nighttime appendicectomy in a cohort of 1,198 patients and suggested that emergency appendicectomy should be performed in the next available emergency theatre rather than delayed solely because surgery occurs outside routine working hours [7]. A large Swedish retrospective cohort study involving 4,950 patients also reported comparable postoperative complications, readmission rates, mortality and length of hospital stay across different operative periods [9].

The WSES Jerusalem Guidelines recommend appendicectomy within 24 hours for most clinically stable patients rather than restricting operative management to daytime hours [1]. Collectively, these studies suggest that operative timing may not be independently associated with adverse short-term outcomes when emergency appendicectomy is delivered within established emergency surgical pathways [1,4,7-9].

Our findings are broadly consistent with this literature. Baseline age, ASA Physical Status Classification and imaging-defined complicated appendicitis were comparable between the IH and OH cohorts. No statistically significant differences were identified in postoperative complications, length of stay or 30-day hospital re-presentation. These findings should, however, be interpreted as an absence of detected differences rather than evidence of equivalence or non-inferiority between the two operative periods.

Access to theatre

The OH cohort had a lower observed mean time from radiological confirmation to commencement of surgery than the IH cohort (16.0 ± 14.0 vs 22.9 ± 24.4 hours); however, this difference was not statistically significant (p = 0.073). The observed difference may reflect variation in emergency theatre availability, prioritisation of urgent cases and competition with elective operating during routine working hours. These factors could not be independently evaluated within the present study.

The findings highlight the complexity of interpreting operative timing in emergency appendicectomy. The time at which surgery occurs is closely linked to service-level factors including theatre capacity, staffing, consultant availability and case prioritisation. Consequently, differences in time to theatre cannot necessarily be attributed to the operative period itself. Further multicentre studies incorporating detailed information on theatre availability, staffing and case prioritisation would help clarify these relationships.

Postoperative complications

Overall, 23/127 (18.1%) patients experienced at least one postoperative complication. Intra-abdominal collection was the most frequently observed complication, occurring in 11/127 (8.7%) patients. Other complications included pain requiring review, postoperative ileus, postoperative fever and wound leak. Two cases involving a retained appendicolith were managed as intra-abdominal collections with antibiotics and were therefore included within this category.

The observed frequency of intra-abdominal collections is consistent with previous literature identifying intra-abdominal abscess formation as an important postoperative complication following complicated appendicitis and appendicectomy [1,6,10]. Previous studies have reported considerable variation in the incidence of postoperative intra-abdominal abscess. In a cohort of 1,817 patients undergoing laparoscopic appendectomy, the overall incidence of postoperative intra-abdominal abscess was 1.5%, with peritoneal irrigation identified as the principal risk factor in the overall cohort [10]. Differences in patient selection, disease severity and definitions of intra-abdominal collection are likely to contribute to variation between reported rates.

In the present study, all intra-abdominal collections were managed with antibiotics alone, and no patients required image-guided drainage or further operative intervention. Postoperative complications were classified according to the Clavien-Dindo classification system based on the treatment required. No Grade III-V complications occurred. Overall, the postoperative complications observed in this cohort were managed conservatively or with pharmacological treatment, without radiological intervention, further surgery or critical-care management.

Conversion to open surgery

All procedures were commenced laparoscopically, with two patients requiring conversion to open right hemicolectomy because of severe complicated appendicitis with caecal involvement. This represented a conversion rate of 2/127 (1.6%). In comparison, a 15-year single-centre analysis of 2,193 adult patients undergoing laparoscopic appendicectomy reported 52 conversions to open surgery (2.4%), with complicated appendicitis and peritonitis identified as independent risk factors for conversion [11].

Both conversions in the present study occurred during IH operating with consultant surgeons present and were associated with severe underlying disease, including caecal involvement and extensive peritonitis. These cases therefore appear more plausibly related to the severity and anatomical extent of the disease than to operative timing.

Limitations

This study has several limitations. First, its retrospective single-centre design may limit the generalisability of the findings to institutions with different emergency surgical pathways, staffing models and theatre provision. As an observational study, it can identify associations but cannot establish a causal relationship between operative timing and clinical outcomes.

Second, operative timing may have been influenced by several potential confounding factors, including theatre availability, staffing levels, consultant supervision, case prioritisation, surgeon experience and patient- or disease-level characteristics. Although age, ASA Physical Status Classification and imaging-defined disease severity were comparable between cohorts, residual confounding cannot be excluded.

Third, the OH cohort encompassed a broad period from early evening through to the early hours of the morning. The operative environment may differ between procedures performed during the early evening and those undertaken overnight, including differences in staffing, consultant supervision and case selection. The study was not designed to examine these subgroups separately.

Fourth, the relatively modest sample size may have limited statistical power to detect differences in uncommon outcomes. In particular, the observed difference in time to theatre should be interpreted cautiously, as the Mann-Whitney analysis did not demonstrate a statistically significant difference (p = 0.073). Similarly, the absence of statistically significant differences in postoperative complications, length of stay and 30-day hospital re-presentation should not be interpreted as demonstrating equivalence or non-inferiority between IH and OH surgery.

Fifth, no formal prospective sample-size or power calculation was performed because all eligible patients during the predefined study period were included. The study may therefore have been underpowered to detect small differences between cohorts.

Finally, only short-term postoperative outcomes were assessed, and surgeon experience and trainee grade were not systematically recorded. Larger multicentre studies incorporating detailed assessment of staffing, theatre availability, case prioritisation and patient- and disease-level factors are required to further evaluate the association between operative timing and outcomes following emergency appendicectomy.

## Conclusions

Out-of-hours appendicectomy was associated with a shorter observed time from radiological confirmation to commencement of surgery compared with IH appendicectomy, although this difference did not reach the predefined threshold for statistical significance (p = 0.073). No statistically significant differences were observed between cohorts in postoperative complications, length of hospital stay or 30-day hospital re-presentation rates. Within this cohort, no difference in the measured short-term clinical outcomes was detected according to operative timing. However, the retrospective observational design and relatively small sample size limit the extent to which these findings can be interpreted as demonstrating equivalence between IH and OH surgery.

Future multicentre studies with larger patient cohorts should evaluate whether outcomes differ between early evening and overnight appendicectomy while accounting for theatre availability, staffing, consultant supervision, case prioritisation and patient- and disease-level factors. Further research should also investigate patient, disease and operative factors associated with postoperative intra-abdominal collections, which were the most frequently observed postoperative complication in this cohort.
